# The Danger of Tea Drinking

**Published:** 1890-04

**Authors:** 


### THE DANGER OF TEA DRINKING.

It is a great misfortune that the popular name for a person who abstains from all alcoholic liquors is a teetotaler ; the term has fostered the idea that tea is a harmless beverage, and it is no doubt true that the moderate use of well made and not very strong tea is less harmful than the habitual resort to any other stimulant. When, however, tea drinking ceases to be the amusement of the leisure moment of a busy afternoon, and is resorted to in large quantities and strong infusions as a means of stimulating the flagging energies to accomplish the alloted task, then distinct danger commences. A breakdown may ensue in more than one way ; not infrequently the stimulus which tea in time fails to give us is sought in alcohol, and the atonic flatulent dyspepsia which the astringent decoction made by long drawing induces, helps to drive the victim to seek a temporary relief in spirits, sal-volatile or even eau de Cologne, which is at first dropped on sugar and finally drank out of a wine glass. In other cases, by ladies especially, relief is sought from morphine, and in a predisposed person the morphine habit is established with extraordinary rapidity. It has been said that as long as a person takes stimulants simply for their taste he is comparatively safe, but as soon as he begins to drink for the effect, then he is running into danger. This is, perhaps, to state the case for stimulants rather too favorably, but if the rule were adhered to we should have fewer cases of educated people sliding into habits of secret intemperance or into morphinomania.—*British Medical Journal.*

